# Functional exercise after total hip replacement (FEATHER) a randomised control trial

**DOI:** 10.1186/1471-2474-13-237

**Published:** 2012-11-28

**Authors:** Brenda Monaghan, Tim Grant, Wayne Hing, Tara Cusack

**Affiliations:** 1 Department of Physiotherapy, Our Lady’s Hospital, Navan, Co Meath, Republic of Ireland; 2 Centre for Support and Training in Analysis and Research (CSTAR), School of Public Health, Physiotherapy and Population Science, Woodview House, University College Dublin, Belfield, Dublin 4, Ireland; 3 Head of Department, Faculty of Health Sciences and Medecine, Bond University, Robina, QLD 4229, Austalia; 4 Physiotherapy and Population Science, School of Public Health, University College Dublin, Belfield, Dublin 4, Ireland

**Keywords:** Total hip replacement, Late stage exercise, Functional exercise, Physiotherapy

## Abstract

**Background:**

Prolonged physical impairments in range of movement, postural stability and walking speed are commonly reported following total hip replacement (THR). It is unclear from the current body of evidence what kind of exercises should be performed to maximize patient function and quality of life.

**Methods/design:**

This will be a single blind multi centre randomized control trial with two arms. Seventy subjects post primary total hip arthroplasty will be randomized into either an experimental group (n=35), or to a control group (n=35). The experimental group will attend a functional exercise class twice weekly for a six week period from week 12 to week 18 post surgery. The functional exercise group will follow a circuit based functional exercise class supervised by a chartered Physiotherapist. The control group will receive usual care. The principal investigator (BM) will perform blinded outcome assessments on all patients using validated measures for pain, stiffness, and function using the Western Ontario and Mc Master Universities Osteoarthritis index (WOMAC). This is the primary outcome measurement tool. Secondary outcome measurements include Quality of life (SF-36), 6 min walk test, Visual Analogue Scale, and the Berg Balance score. The WOMAC score will be collated on day five post surgery and repeated at week twelve and week eighteen. All other measurements will be taken at week 12 and repeated at week eighteen. In addition a blinded radiologist will measure gluteus medius cross sectional area using real time ultrasound for all subjects at week 12 and at week 18 to determine if the functional exercise programme has any effect on muscle size.

**Discussion:**

This randomised controlled trial will add to the body of evidence on the relationship between muscle size, functional ability, balance, quality of life and time post surgery in patients following total hip arthroplasty. The CONSORT guidelines will be followed to throughout. Ethical approval has been gained from the Ethics committee Health Services Executive Dublin North East.

**Trial registration:**

This trial is registered with ClinicalTrials.gov (a service of the United States National Institutes of Health) identifier NCT01683201

## Background

Total hip replacement (THR) is a very common surgical procedure carried out worldwide. Approximately 24,253 THR surgical procedures were performed in Canada in 2008/09 [1] with almost one million hip and knee replacements undertaken in the U.S in the same time-frame. [2] In England and Wales 53,462 hip replacements were undertaken in 2009 including both primary and revision joint procedures [3]. These figures have been projected to grow with the aging population and with the increased prevalence of arthritis in the elderly [4]. Improvements in prosthetic design and higher community expectations for quality of life have made THR more appropriate for both older and younger age groups resulting in an increased population and healthcare demand for surgery [5].

In recent years the shift towards reductions in the length of stay for patients following THR has caused a subsequent shift in rehabilitation services from ’in hospital’ to out patients [6]. Within the outpatient setting however a number of authors have reported functional limitations for patients post surgery. Pain, physical impairment, reduced range of motion and reduced muscle strength persisting for one year have been documented in subjects who had also received early stage physiotherapy [7]. It is currently unclear from the literature which therapeutic exercises performed over what period of time are either effective or necessary to improve muscle strength or ensure optimal return of patient function. Anecdotally therapeutic exercise programmes appear to be largely based on clinical experience and surgeon preference [8]. Some evidence [9] has demonstrated that an eight week exercise programme with an emphasis on strengthening and stability resulted in a statistically significant improvement in all measurements of self perceived function and muscle strength in patients between four and twelve months post THR. It has been suggested in this study that patients should continue their functional strengthening exercise programme for at least one year and that this programme should be further progressed as their function improved.

Another study [10] compared differences in the isometric hip strength of patients five months after THR with older adults without hip surgery and demonstrated lower peak torque in the hip flexors of the hip surgery group. Specific rehabilitation programmes are needed to address these strength deficits at this phase post surgery although few studies to date have evaluated this subject.

The gluteus medius muscle is critical to the provision of abduction force across the hip and the strength of the muscle is specifically relevant to the provision of stability in the hip joint [11]. Post operative function is determined by regaining the optimal strength in this muscle and many rehabilitation programmes include strengthening of gluteus medius. The strength of this muscle provides lateral stability to the trunk and pelvis and is essential during one legged stance and also during the stance phase of gait [12]. However there is a paucity of evidence in the literature examining specific changes in this muscle despite anecdotal evidence that reduced strength in this muscle is responsible for gait impairment and reduced balance [13].

Recognizing the difficulties in measuring rehabilitation of glutei dysfunction it has been suggested that future studies on the glutei muscles should evaluate each muscle in functional activities preferably using multiple parameters for example evaluation of muscle size together with functional assessment [14]. Recently real time ultrasound imaging has been utilized as an accurate and reliable means of measuring muscle parameters in a non invasive manner [15,16]. Ultrasound imaging is a fast and inexpensive tool that produces excellent images of the musculoskeletal system without the need for radiation [17].

The primary aim of this research project is to evaluate the efficacy of a specific functional exercise programme to improve pain stiffness and physical function in patients between 12 and 18 weeks post THR when compared with a group following usual care. In addition this project aims to measure the effect of a functional exercise programme on the secondary outcomes of quality of life, balance, function and muscle size. Muscle size measured by real-time ultrasound was selected as an additional secondary outcome in order to examine specific changes in the gluteus medius muscle.

In preparing this protocol a full systematic review and meta-analysis of previous work has been completed. This review found there was low grade evidence that late stage rehabilitation demonstrated significant improvement in gait speed indicating improved walking ability in patients post total hip arthroplasty and there was limited low grade evidence for improved hip abduction strength in patients following a programme which included low resistance strength training. However the full extent of the impact of late stage rehabilitation in patients post total hip arthroplasty has not yet been established. These issues were considered in designing this randomized controlled trial.

Other preparatory work to prepare this protocol included preliminary clinical patient evaluation in a single elective orthopedic centre. A telephone questionnaire of 120 patients in 2009 at 12 weeks post surgery demonstrated approx 30 % of patients contacted reported persistent limping and gait abnormalities at 12 weeks. These patients were not previously referred to Physiotherapy. A second telephone survey evaluated ten centers nationally which carry out elective hip arthroplasty and demonstrated a wide diversity of rehabilitation offered across the country and a paucity of evidence based practice guidelines for this client group.

The primary aim of this research project is to evaluate the efficacy of a specific functional exercise programme to improve pain, stiffness and physical function in patients between 12 and 18 weeks post total hip replacement when compared with a group following usual care. In addition this project aims to measure the effect of a functional exercise programme on the secondary outcomes of quality of life, balance, function and muscle size. Muscle size measured by Real time ultrasound was selected as an additional secondary outcome measuring specific changes in the gluteus medius muscle. There is a paucity of evidence in the literature examining specific changes in this specific muscle despite anecdotal evidence that reduced strength in this muscle is responsible for gait impairment and reduced balance.

### Primary hypotheses

A six week functional exercise programme from week 12 to week 18 in patients post total hip replacement will be more efficacious in improving pain, stiffness and function than the usual care programme.

### Secondary hypotheses

A six week functional exercise programme from week 12 to week 18 in patients post total hip replacement will be more efficacious in improving balance, gait speed, quality of life and muscle size than the usual care programme.

## Methods/design

### Trial design

This will be an assessor blinded two arm randomized controlled trial of a six week involving twelve functional exercise class interventions. Measurements will be taken at day five, week twelve and week eighteen for all subjects. Assessment of adherence to the treatment programme will be assessed with examination of exercise logs completed by clients at each attendance and signed off by the supervising therapist. The classes will be conducted at three sites, Navan, Cavan and Monaghan. The protocol will conform to CONSORT guidelines for reporting non-pharmalogical interventions and has been registered with clinical trials.gov prior to study commencement.

### Participants

We will recruit participants at pre-assessment for primary total hip replacement at Our Lady’s hospital, Navan which is the elective joint replacement for the Louth/Meath and Cavan/Monaghan areas. To be eligible the following inclusion and exclusion criteria must be met.

### Inclusion criteria

Patients 12 weeks post primary unilateral anterior approach THA for osteoarthritis.

Age 50 years and above.

Able to read and understand instructions in English.

Willing to attend classes twice weekly for 6 weeks

Able to participate in an exercise programme without physical assistance.

Mobilizing independently 15m without crutches and/or stick and passed by the referring Orthopaedic consultant as suitable for inclusion in the study at the routine six week post surgery appointment.

### Exclusion criteria

Medically unstable.

Have central or peripheral nervous system deficits.

Any underlying terminal disease.

Suspicion of infection following joint replacement.

### Procedure

Preliminary screening will be carried out at the pre-assessment clinic by the principal investigator (BM). Following a standard interview process patients will be given a written description of the study and information with regard to the exercise class commitment involved. A screening record will be kept to document criteria eliminating those deemed to be ineligible. All patients will all be assessed using the WOMAC questionnaire on day 5 prior to discharge and they will be randomized to one of two treatment groups. The functional exercise intervention begins at week 12 and all patients will complete the Primary and Secondary outcome assessments at this point. All assessments are repeated for both groups at week 18. Patients allocated to the functional exercise class will attend a six week functional exercise programme twice weekly from week 12 to week 18. The control group will receive the standard usual post surgery care as outlined in the patients’ information booklet given to all patients on admission. This care is given to all patients post surgery by the Physiotherapist working on the ward. This study is one comparing usual treatment with an intervention of functional exercise. All patients randomised to the usual care group will be offered the chance to attend the exercise group at week 18 post evaluation.

### Ethical considerations

This study obtained ethical approval from the HSE Research Ethics Committee Dublin North East Area in December 2011 for the clinical sites at Our Lady’s Hospital, Navan, Cavan General Hospital and Monaghan General Hospital.

### Blinding

Both outcome assessors will be blind to group allocation and will not be involved in the interventions nor will they attend any of the functional exercise classes. The physiotherapists supervising the functional exercise classes are not blinded. The statistician will be blinded to group allocation prior to completion of the statistical analysis.

### Randomisation and allocation concealment

Following a standard interview at the pre assessment clinic, Patients will be given a written description of the study and information with regard to the exercise class commitment involved. They will also be given stamped addressed envelopes to post back written consent. Following obtainment of written consent random allocation using sequentially numbered envelopes to both groups will be carried out by a third party Lara Bourton Cassidy (LBC) who is not involved in the study. The number sequence will be generated using a computer generated random number table generated by Dr Tim Grant and communicated directly to LBC. Subjects will be randomly allocated either to a functional exercise class from week 12 post op to week 18 or to a control group receiving usual post operative care. Allocation of patients to the treating therapists will be established by e mail contact from LBC to the Physiotherapist’s responsible for conducting the classes. These therapists have been identified previously. Patients will be asked not to discuss their group allocation and all blinded outcome assessment examinations will take place in Our Lady’s Hospital, Navan.

### Physiotherapists

Three experienced physiotherapists one at each site will be trained to supervise the functional exercise classes. Training will comprise a one hour practical workshop and all physiotherapists will be provided with a written illustrated manual of the training workshop.

### Exercise interventions

This functional exercise class is designed to strengthen the hip muscles and incorporates exercises commonly used in clinical practice. It is based on previous work that shows that such an exercise programme improves pain and function [18]. Twelve exercises aimed to strengthen the quadriceps, hamstrings, and hip abductor muscles and improve functional balance will be taught to participants by the Physiotherapist. See Table 1. At the end of the exercise session the physiotherapist will monitor proper form and exercise intensity and will progress the exercises as necessary. Each Physiotherapy session will be 30 minutes in length. Intensity will be determined by the participant’s ability to complete 10 repetitions for a given exercise.

**Table 1:** Functional Hip Exercise circuit

**Exercise**	**Description and progression**
Sit to stand	Sit to stand exercise from chair. Hands used to guide only. Progress with increased repetitions and speed.
Toe raises	Stand straight feet flat on floor, Keep abdomen tight and hold one leg up as you raise on toes as high as possible.
Knee raises	Back straight slowly lift one leg as high as you can and lower to floor raise opposite arms as knee is raised.
Side and back leg raises	Slowly raise leg out to side and then return to start. Repeat with opposite side. Repeat exercise with leg into extension.
Partial knee bends	Stand on both legs. Keep abdomen tight lower towards floor by bending knees to approx 30 degrees. Straighten knees and repeat.
Single knee bends	Stand on one leg, grip floor with toes and slowly lower to approx 30 degrees progress with dumbbells.
One legged standing balance	Use two chairs transfer weight to operated leg and pick other leg up try to balance for 10 seconds. Progress with timed balance.
Advanced one legged balance	As previous but also turn head from side to side.
Pelvic raising/lowering	Stand on operated leg. Stand unsupported not allowing pelvis to drop. Progress by slowly raising and lowering pelvis on the side of the bent knee.

### Follow-up period

The duration of this research project is eighteen weeks from the date of surgery. All patients will complete the WOMAC questionnaire on discharge from hospital at day 5 and all outcome measurements will be repeated at week 12 and again at week 18. The outcome measurement assessments at week 12 and at week 18 will take place in Our Lady’s Hospital in Navan.

### Measurements

Baseline descriptive data will be obtained by questionnaire and will include age, sex, weight, height, medication use and previous health problems. A summary of all measurements is included in Table 2

**Table 2:** Summary of outcomes to be collected

**Primary Outcome Measurement**	**Data Collection Instrument**	**Collection times in weeks**
Pain	Pain Subscale of the WOMAC osteoarthritis index 3.1 Likert Version	Day 5 Week 12 Week 18
Self reported Physical Function	Physical Function subscale of the WOMAC osteoarthritis index 3.1 Likert Version	Day 5 Week 12 Week 18
Self reported Stiffness	Stiffness Function subscale of the WOMAC osteoarthritis index 3.1 Likert Version	Day 5 Week 12 Week 15
**Secondary Outcome measurement**		
Real time ultrasound of the Gluteus Medius Muscle	Cross sectional area measurement	Week 12 Week 18
6 Minute Walk Test	Functional Performance	Week 12 and Week 18
Short Form SF-36	Self reported measurement of health Status and quality of life	Week 12 and Week 18
Berg Balance Score	Functional balance assessment scale	Week 12 and Week 18
Visual Analogue Scale	Average overall pain in past week 100mm VAS	Day 5, Week 12, and Week 20

### Primary outcome measurements

#### Western Ontario and Mc master universities osteoarthritis index (WOMAC) likert version 3.1

This is a multidimensional, self administered disease specific standardized instrument which is proven to be reliable and valid within this client group [19]. The outcomes will be completed during face to face contact with the assessor (BM) in the Physiotherapy department prior to hospital discharge on day five and repeated at week 12 and week 20. The questionnaire consists of 24 questions regarding pain (scored 0–20) stiffness (scored 0–8) and physical function (scored 0–68). For each division a score is calculated the range of scores is from 0–98 with higher scores indicating a lower health status. Minimal perceptible clinical improvement on the WOMAC questionnaire has been established in the literature at −10.4 with the standard deviation of scores on the instrument at 13.6 [20].

#### Secondary outcome measurements

##### Real time ultrasound imaging of the gluteus medius muscles

Real time ultrasound of the gluteus medius will establish the size of the muscle pre and post the functional exercise class. Measurement on day five is not possible due to wound dressing and patient positioning issues. Patients will be scanned by the radiologist at week 12 and the scan will be repeated at week 18. Intra therapy reliability testing will be carried out prior to scanning and the radiologist will be blinded to group allocation. Repeated measurement at week 18 will allow for comparison of both groups with and without intervention. Exploratory analysis on a subgroup of patients using real timed ultrasound to assess the size of gluteus medius in the non affected side will also carried out. This subgroup will be randomly selected from the main group following initial randomisation. It is planned to compare the size of the gluteus medius in the non affected side with the affected side at week 12 and again at week 18.

##### 6 Minute walk test

This is a reliable and valid test of physical function in this client group and has been shown to be responsive to detecting deterioration and improvement in the early post operative period [21]. It will be completed by the principal investigator (BM) who will remain blind to group allocation.

##### Short form SF-36

This is a well recognized and valid self reported questionnaire that measures health status and quality of life [22]. It will be completed in the presence of the principal investigator who will remain blinded to group allocation.

##### Berg balance score

This assessment scale of ability to maintain balance both statically and whilst performing various functional tasks will be completed by the blinded principal investigator (BM) at week 12 and repeated for all patients at week 18. It has been demonstrated to be valid and reliable in older adults [23].

##### Visual analogue scale

This 100mm scale with descriptors at either end of the scale of “no pain”, and “worst possible pain” allows for self assessment of levels of pain at any specific time. It has been demonstrated to be reliable in the osteoarthritic client group [24].

#### Sample size

The power calculations were based on the physical function subscale of the WOMAC questionnaire. The minimal clinical difference on the WOMAC questionnaire has been established in the literature at −10.4 with the standard deviation of scores on the instrument at 13.6 [20]. The sample size was then calculated requiring a power of 80% in a two tailed test with a significance level of .05. The effect size was calculated at 10.4/13.6. It was .764 or a mod/large effect size. The sample size required was then calculated at 27 patients in each group or 54 in total. This would increase to 60 patients in total allowing for 10% attrition.

Consideration of the power calculations based on both the Berg balance score and the SF36 short form health survey, similar numbers are required. Using minimal detectable change scores and standard deviations from the literature [25] sample sizes at 80% power and .05 significance in a two tailed test were calculated at 70 subjects in total for the Berg Balance score and 54 subjects for the SF36 respectively. Therefore 70 subjects in total or 35 in each group would allow for 10% attrition for all outcome measurement tools.

#### Statistical analysis

Data analysis will be performed in consultation with Mr. Tim Grant in CSTARS. The primary conclusions of this project will be based on analyses conducted under the principal of intention to treat. All randomised patients will be analysed in the groups to which they are originally allocated to regardless of whether they actually received the intended treatment or not. Missing primary outcomes will be assumed to be missing at random and will not be considered in the primary analysis.

Primary analysis of the effect of functional exercise will incorporate continuous variables which are expected to be normally distributed. These will be presented as mean and standard deviations and will be assessed using t tests at different time points i.e. at 12 weeks and again at 18 weeks. If the data is not normally distributed it is planned to use the non parametric Wilcox on signed ranks test.

#### A priori defined subgroup analysis

Exploratory analysis on a subgroup of patients using real timed ultrasound to assess the size of gluteus medius in the non affected side will be carried out. This subgroup will be randomly selected from the main group following initial randomisation. It is planned to compare the size of the glut medius in the non affected side with the affected side at week 12 and again at week 18. This data is expected to also to be normally distributed and will be assessed using t tests at the various time points. The results of any subgroup analyses will be reported and submitted for publication soon after the primary publication.

### 2.5 Data set issues

The trial database will be frozen by the principal investigator (BM). The statistician (TG) will work on a copy of the trial database that has been downloaded to them by the principal investigator.

Any amendments to the data set will be documented.

The statistical software used for the analysis will depend on the preference of the statistician or person doing the statistical analysis based on what is widely used.

#### Timelines

This study has been funded by the Health Research Board and ethics approval has been obtained from the ethics board of the HSE Dublin North East branch in January 2012. Recruitment and training of Physiotherapists will occur in October/November 2012 and the anticipated timelines for this project are as follows.

September 2012; Recruitment commences

October/November; Physiotherapist training

January 2013; Participants begin pre intervention testing

January 2014; All participants completed intervention and post intervention follow up.

## Discussion

This research project will focus on the effectiveness of a specific functional exercise programme to prevent residual hip muscle weakness and subsequent disability following elective surgery and it also will incorporate the use of a real time ultrasound to measure the effectiveness of functional exercise on muscle atrophy in the short term. Specifically the patient group now requiring joint replacement in Ireland as in the rest of the developed world comes from that demographic bulge in the population previously dubbed the ‘baby boomer’ generation. Need for joint replacement surgery together with their increased expectation of quality of life and the economic reality of shorter inpatient length of stay will shape reformation of rehabilitation needs post total hip replacement for this group in the future.

This research project aims to assess the effect of a functional exercise programme in the THR patient group incorporating specific assessment of balance, quality of life and gluteus medius size using real time ultrasound as the secondary outcome measurements. This is very relevant to the specific patient population post total hip replacement. It is critical to the improvement of health and social gain in terms of improving post surgical functional ability and the subsequent ability of people following THR to contribute to society in a meaningful way.

## Competing interests

The authors declare they have no competing issues.

## Authors’ contributions

All authors contributed to the development and writing of the protocol. All authors have been involved in the drafting and revision of this manuscript and have given their approval for the final version.

## Pre-publication history

The pre-publication history for this paper can be accessed here:

http://www.biomedcentral.com/1471-2474/13/237/prepub
